# Treatment Options for Massive Irreparable Rotator Cuff Tears

**DOI:** 10.1007/s12178-021-09714-7

**Published:** 2021-09-28

**Authors:** Natalie K. Kucirek, Nicole J. Hung, Stephanie E. Wong

**Affiliations:** 1 School of Medicine, University of California, San Francisco, San Francisco, CA USA; 2 Department of Orthopaedic Surgery, David Geffen School of Medicine at UCLA, Los Angeles, CA USA; 3 Department of Orthopaedic Surgery, University of California, San Francisco, 1500 Owens Street, San Francisco, CA 94158 USA

**Keywords:** Massive rotator cuff tear, Irreparable rotator cuff tear, Reverse total shoulder arthroplasty, Superior capsule reconstruction, Subacromial balloon spacer, Rotator cuff repair

## Abstract

**Purpose of Review:**

Massive irreparable rotator cuff tears present a significant challenge to the orthopedic surgeon. No single treatment, particularly among joint-preserving options, has been shown to be superior. The purpose of this review is to discuss recent advances in the treatment of massive irreparable rotator cuff tears, including partial repair with and without graft augmentation, interposition grafts, superior capsule reconstruction, subacromial balloon spacers, tendon transfer, and reverse total shoulder arthroplasty. We will also offer guidance on surgical indications based on our clinical experience.

**Recent Findings:**

Partial repair may offer reasonable clinical improvement for patients with lower preoperative function despite high re-tear rates. Additionally, several types of interposition grafts have shown promising short-term results and may outperform repair alone. Subacromial balloon spacers may lead to clinical improvement, especially in patients without glenohumeral osteoarthritis or pseudoparalysis, and recently received FDA approval for use in the USA. Superior capsule reconstruction is a technically demanding procedure that appears to produce excellent short-term results particularly when performed at high volume, but long-term studies in heterogeneous study groups are needed. Tendon transfers improve function by restoring force coupling in the shoulder, offering a promising option for younger patients. Reverse total shoulder arthroplasty (RTSA) is a reliable option for treatment of irreparable cuff tears in elderly patients with lower functional demands.

**Summary:**

Irreparable cuff tears remain a difficult condition to treat. Recommended treatment for younger patients without glenohumeral osteoarthritis is particularly controversial. For older patients with low-demand lifestyles and glenohumeral osteoarthritis, RTSA is an effective treatment option. For all discussed procedures, patient selection appears to play a critical role in clinical outcomes.

## Introduction

Rotator cuff tears rank among the most common musculoskeletal injuries encountered by orthopedic surgeons, with a reported prevalence of 20% in the general population and 30% in cadaveric studies [1, 2]. Rotator cuff pathology is most often due to age-related degeneration, with studies reporting tears in up to 54% of those over 60 and 62% of those over 80 years old, but can also result from traumatic injury such as shoulder dislocation [1, 3–5]. Among rotator cuff tears, massive tears present a particular challenge due to high rates of re-tear ranging from 18 to 94% in recent studies, failure of healing after repair, and the potential for irreparability [6]. Massive rotator cuff tears have been defined by various criteria, including a tear with a diameter of 5 cm or greater by Cofield, detachment of two or more tendons from the tuberosities by Gerber, and a contracted tear greater than 2×2 cm in the coronal and sagittal planes by Davidson and Burkhart [7–9]. Given these disparate classifications, Schumaier et al. convened an expert group to generate a consensus definition of massive cuff tears, which they defined as tears with retraction to the glenoid rim in the axial or coronal planes, or with at least two-thirds of the greater tuberosity exposed in the sagittal plane [6].

Clinically, patients with massive cuff tears may be asymptomatic. When symptomatic, patients often have pain, classically at night and with overhead motion, although the degree of pain has not been shown to correlate with tear severity [10]. Other symptoms include weakness and impaired active range of motion with physical examination findings correlating with location of the tear. Posterosuperior tears involving the supraspinatus and infraspinatus often produce weakness in external rotation, whereas anterosuperior tears involving the subscapularis may lead to a positive lift-off test.

While many massive rotator cuff tears are treatable with repair, some are determined to be irreparable, although this designation remains controversial given multiple factors that contribute to irreparability. Previous studies defined an irreparable rotator cuff tear by the presence of cuff tendons so contracted or atrophied that they cannot be reattached to the footprint during mobilization and attempted repair [11]. Importantly, “massive” and “irreparable” are not synonymous descriptors; while most irreparable tears are massive, some are not, and many massive tears are reparable. The concept of “predictive irreparability” posits that preoperative imaging and clinical characteristics can predict whether a tear will be reparable or not. Findings associated with irreparability in the literature include a positive tangent sign on magnetic resonance imaging (MRI), Goutallier grade 3–4 fatty infiltration of the supraspinatus, an acromiohumeral interval less than 6 mm, superior migration of the humeral head, a U-shaped tear, and chronic pseudoparalysis [12–16]. Other patient- and tear-related factors, such as smoking, older age, diabetes, chronicity of tear, and limited preoperative active range of motion, may also portend failure of primary repair [17]. However, most argue that all patients should undergo arthroscopic examination and attempted repair before the surgeon deems a tear irreparable, rather than relying on predictive factors to make treatment decisions [13, 18].

Treatment options for massive irreparable rotator cuff tears are numerous and growing, but no treatment has been deemed superior [11]. When nonsurgical options such as physical therapy, NSAIDs, and injections fail to provide adequate pain relief and functional restoration, surgical options may be considered. In this review, we aim to provide a current update on the landscape of surgical treatment for massive irreparable rotator cuff tears. We will examine the latest evidence for partial repair with and without graft augmentation, interposition grafts, subacromial balloon spacers, superior capsule reconstruction, tendon transfer, and reverse total shoulder arthroplasty (RTSA). We will also offer guidance on indications for the discussed treatment options of these challenging injuries based on our clinical experience and analysis of the current literature.

## Partial Repair

The concept of partial repair of massive irreparable rotator cuff tears was introduced by Burkhart in 1994. The goal of partial repair is to restore force couples in the shoulder by repairing the anterior and posterior cuff, leaving a “functional rotator cuff tear” in the retracted superior cuff. Per Burkhart, this repair creates a biomechanically, though not anatomically, intact rotator cuff [19]. Postoperative re-tear rates after partial repair are high, with a recent systematic review reporting a structural failure rate of 48.9% across four studies [20]. Given high re-tear rates, a common concern is that partial repair outcomes may not be durable, and that short-term benefits may be a result of concomitant adjuvant techniques commonly performed such as subacromial decompression, debridement, bursectomy, and biceps tenotomy or tenodesis, rather than of the partial repair itself.

Despite the high reported re-tear rates, studies have shown high patient satisfaction, reasonable functionality, and low rates of revision surgery at mid-term follow-up after partial repair for irreparable rotator cuff tears. Galasso et al. demonstrated an improvement in Constant scores from 39.1 ± 8.4 to 76.3 ± 9.7 (*p <*0.001) at a mean of 7 years, with 87.4% of patients reporting complete satisfaction [21]. Hallock et al. found a 5.2% rate of revision surgery at 4.5 years, with 87% of patients requiring no additional treatment [22]. Besnard et al. saw improvements in Constant scores from 31.0 ± 9.2 to 77.1 ± 13.0 at first follow-up 2–5 years after surgery, although scores decreased slightly to 72.8 ± 14.1 after 7–10 years (*p*=0.006) [23•]. On the other hand, Shon et al. reported nearly 50% of patients felt nearly the same or dissatisfied at mean follow-up of 24 months, with a decline in ASES scores in the “same or dissatisfied” cohort over the course of 12 to 24 months postoperatively (*p*<0.05) [24].

Partial repair has also performed comparably when compared to more aggressive treatment strategies. Jeong et al. retrospectively compared patients who underwent partial repair with margin convergence to those whose tears were made reparable through a posterior interval slide [25•]. After an average of 84.1 months, there were no statistically significant differences in clinical outcome measures or range of motion, suggesting that utilization of aggressive techniques to make an irreparable tear “reparable” is not superior to a more conservative partial repair. A retrospective cohort comparison study between partial repair and latissimus dorsi tendon transfers (LDTT) for massive rotator cuff tears also reported no significant difference between groups in postoperative functional scores, active forward elevation, or external rotation at 2 years [26].

When considering candidates for partial repair, patients with worse preoperative function may have more to gain from a partial repair. Chen et al. found that patients with lower preoperative American Shoulder and Elbow Surgeons (ASES) scores, higher visual analog scale (VAS) pain scores, and night pain had greater improvement in ASES scores 2 years after partial repair [27]. On the other hand, preoperative stage 2 or higher fatty infiltration of the teres minor has been associated with worse outcomes after partial repair [24, 26]. In summary, partial repair is a joint-preserving procedure that often leads to functional improvement in at least the short and mid-term and may be most beneficial for patients with low preoperative functional status. While re-tear is common, it may not necessitate re-operation, and favorable clinical outcomes are still possible [25•].

## Repair with Augmentation and Interposition Grafts

In order to achieve repair of irreparable cuff tears, surgeons have also utilized xenograft, allograft, autograft, and synthetics for augmentation or interposition grafting. In partial repair with augmentation grafting, the rotator cuff tendon is repaired in partial fashion, then a graft is added over the tendon and covering the remainder of the foot print with the goals of improving stability and/or biologic healing. In contrast, interposition grafts bridge the gap between an irreparable rotator cuff tear and the footprint, and are attached to the retracted cuff tendon medially and the tuberosity laterally, enabling a low-tension repair [28, 29]. A recent systematic review and meta-analysis of augmentation and interposition grafts for rotator cuff tears found that these techniques outperformed repair alone with respect to re-tear rate and ASES scores. However, the authors noted that not all included studies involved massive and irreparable tears exclusively [28].

Recent studies examining the utility of interposition grafts specifically for irreparable massive tears have shown promising results at short- and mid-term follow-up across graft types. Kim et al. reported an improvement in mean ASES scores from 50 preoperatively to 83 (*p*<0.001) at 3 years using human acellular dermal matrix allograft, with no complications or infections [30]. Using acellular porcine dermal matrix xenograft, Neumann et al. reported an improvement in modified ASES scores to an average of 87.8 at 50.3 months, with 91.8% of grafts intact per ultrasound evaluation [31]. Seker et al. reported similar success using polytetrafluoroethylene (PTFE) synthetic felt, with an average ASES score of 95 and Constant score of 90 at 3 years of follow-up and 90% of patches intact [32]. However, long-term evaluations of interposition grafts are limited. A small retrospective case study of 13 consecutive patients who underwent polyester synthetic interposition grafting found that 75% of patients developed cuff-tear arthropathy after 18 years of follow-up, with a 70% failure rate of the graft and an average Constant score of 46 [33]. While this study is small, it raises important concerns about the long-term durability of interposition grafting.

## Subacromial Balloon Spacers

The subacromial balloon spacer was first described in 2012 by Savarese and Romeo for treatment of massive irreparable rotator cuff tears [34]. It has gained significant popularity in Europe and was recently approved by the Food and Drug Administration (FDA) in the USA. InSpace (Stryker, Michigan, USA), the subacromial spacer currently used, is composed of a poly-lactide and ε-caprolactone polymer that is implanted in the subacromial space and then filled with saline, with the intention of increasing, or normalizing, the distance between the humeral head and acromion and well as reducing the friction between the humeral head and acromion. The spacer eventually dissolves after 12 months [34]. A biomechanical study by Lobao et al. demonstrated that after implantation into cadaveric shoulders with irreparable tears, the balloon spacer increased the acromiohumeral interval, lowered the humeral head, increased deltoid loading, and restored glenohumeral contact pressure levels similar to those of an intact shoulder, which may lead to clinical improvement [35]. Its long-term mechanism of action remains uncertain, given its dissolution after 12 months. Szabo hypothesized that implantation of the spacer may lead to scarring in the subacromial space that acts similarly to superior capsule reconstruction, but further research is required to fully elucidate long-term mechanisms [36].

Clinical studies of the subacromial balloon spacer have generally shown positive results in the short and mid-term, though the study with longest follow-up thus far spans only 5 years [37]. A recent meta-analysis by Liu et al., which includes 10 studies with a total of 270 shoulders, demonstrated a pooled mean improvement in Constant scores at final follow-up (between 3 months and 3 years) of 26.4 points, though heterogeneity between studies was high (*p*<0.00001, I^2^ =61%) [38]. In the meta-analysis, complications were rare and included synovitis and spacer migration; however, a case of symptomatic foreign body reaction has since been reported for the first time by Calvo et al. [39]. Two additional recent studies of subacromial balloon spacer placement have also demonstrated good results after 3 years of follow-up. Familiari et al. found an improvement in Constant score from 27.7 ± 7.4 preoperatively to 77 ± 15 at 36 months (*p*<0.01), with 98% of patients achieving minimal clinically important difference (MCID) and 12% requiring revision surgery. Patient satisfaction was 90% [40]. Piekaar et al.’s prospective study demonstrated durable improvement in pain and functional scores that persisted from 1–3 year follow-up and an 82% satisfaction rate [41]. These short- and mid-term results suggest that the spacer’s beneficial effects may persist after it dissolves.

However, studies comparing the subacromial balloon spacer to other established treatments for massive irreparable cuff tears have not shown its definitive superiority [42–44]. Malahias et al. conducted a matched case-control study of 32 patients examining outcomes after partial repair combined with balloon spacer compared to partial repair alone [43••]. While all patients’ functional scores, pain, and range of motion improved at 12 months and the vast majority of each group met the MCID for Constant score, no statistically significant differences were found between the two groups, though there was a trend towards higher patient satisfaction and functional outcomes in the balloon group. Given the novelty of this device, the question of cost-effectiveness compared to other treatment modalities is also salient. In a comparative cost analysis study, Castagna et al. found that the balloon spacer was superior to partial repair, reverse total shoulder arthroplasty, and conservative treatment for irreparable rotator cuff tears, with an incremental cost-effectiveness ratio (ICER) of 10,000 euros/quality-adjusted life year (QALY), below the $50,000 USD threshold typically considered cost-effective [45]. Additionally, subacromial balloon spacers are technically straightforward to perform compared to other surgical procedures for massive, irreparable rotator cuff tears with potential for cost savings with decreased time spent in the operating room.

There is a clear need for randomized controlled trials to compare subacromial balloon implantation with other methods of treatment for massive irreparable tears. Currently, a randomized adaptive clinical trial in the UK comparing the balloon spacer with debridement (START: REACTS trial) is underway, and a large multi-center randomized controlled trial comparing the balloon spacer to partial repair was recently completed in the USA, though results have yet to be published [46, 47]. These trials will also ideally clarify specific indications for balloon spacers, which may include patients without arthritis who have intact passive range of motion and an intact subscapularis [48].

## Superior Capsule Reconstruction

In 2013, Mihata et al. first described the superior capsule reconstruction (SCR) as a novel treatment for irreparable rotator cuff tears. As originally described, the SCR is achieved by anchoring a thick fascia lata autograft medially at the superior glenoid and laterally at the cuff footprint on the greater tuberosity. The graft is then attached to the infraspinatus tendon and the remaining supraspinatus or subscapularis tendon using side-to-side sutures [49]. Initial results reported by Mihata et al. were excellent, with significant gains in active elevation and functional score improvements comparable to those seen in complete rotator cuff repair, as well as a re-tear rate of only 16.7% at nearly 3 years [49]. Soon after, Hirahara described a technique for SCR using dermal allograft, which has become the more prevalent method in North America [50]. Given promising initial results, interest in SCR has soared, with a 300% increase in SCR publications in 2018 compared to 2016 [51].

Multiple short- and mid-term retrospective studies of SCR with both dermal allograft and fascia lata autograft have shown excellent clinical outcomes, with high functional scores, significant pain reduction, and high rates of graft integrity reported at 1–5 years of follow-up [18, 52–55]. Burkhart et al.’s retrospective review of 41 dermal allograft SCRs demonstrated durable improvements in mean ASES scores from 52 ± 3 preoperatively to 90 ± 1 at 1 year (*p*<0.0001) and 89 ± 2 at final follow-up at 34 months (*p*<0.0001) as well as an 85% rate of graft healing. The authors also reported an unsatisfactory outcome rate of 19%, including two revisions and six patients who failed to meet MCID for ASES [18]. Mihata et al.’s retrospective series of 30 fascia lata SCRs included 5 years of follow-up, the longest reported thus far in the SCR literature, and showed progressive improvement in mean ASES scores from 83.0 ± 16.0 at 1 year to 92.3 ± 10.3 at 5 years (*p*=0.03). While 90% of patients had graft healing, the 10% who experienced graft tear had severe cuff tear arthropathy at 5 years [56••].

However, there have been reports of less exceptional results after SCR. Greiner et al. reported the first matched-pair cohort study comparing xenograft SCR to partial rotator cuff repair for irreparable cuff tears. While both groups saw significant and substantial improvements in functional scores, pain, and range of motion at 2 years, there was no significant difference in improvement between the treatment cohorts [57•]. Lee et al. reported a re-tear rate of 35% at a mean follow-up of 31.2 months after allograft or autograft SCR, with 50% of patients with re-tear requiring additional surgery [58]. Additionally, Woodmass et al.’s retrospective review of dermal allograft SCR had a clinical failure rate of 65%, as defined by reoperation (24% of patients) or failure to meet a set of pain, range of motion, and patient satisfaction thresholds [59]. Interestingly, the rate of failure in an individual surgeon’s first ten SCR cases was 77.3%, which fell to 41.7% in subsequent cases (*p*=0.06), highlighting the importance of the surgeon’s volume and experience in this technically demanding operation.

Patient factors that may influence outcomes after SCR have also been explored to better define indications. The presence of subscapularis atrophy or tear preoperatively has been associated with poorer outcomes after SCR [59–61]. Mihata et al.’s cohort study comparing SCR results in patients with an intact, reparable, or irreparable subscapularis demonstrated inferior strength, range of motion, and functional outcomes in the irreparable subscapularis group at 3 years follow-up, as well as increased infection and graft tear rates. Currently, most surgeons consider either an intact subscapularis or a reparable subscapularis a requirement to undergo SCR. There were no significant differences in postoperative functional scores, range of motion, and strength between patients with an intact versus reparable subscapularis tear, demonstrating the importance of repairing subscapularis during SCR when possible [62•]. Other patient factors associated with higher SCR failure rates included prior shoulder surgery, female sex, and higher degree of fatty infiltration in the infraspinatus [54, 59, 60]. Interestingly, two recent studies demonstrated that SCR may be a viable alternative to total shoulder arthroplasty in patients with irreparable tears and severe pseudoparalysis, with reported improvements in active elevation from 36.7° ± 19.1° to 150.0° ± 36.8° (*p*<0.001) and 27° ± 2° to 159° ± 15° (*p*< .0001), respectively, and reversal of pseudoparalysis in 87% and 90% of patients [63, 64].

Technical factors such as graft material, thickness, and stiffness have also been compared in order to optimize outcomes. Biomechanical studies suggest that thicker or double-layer dermal allografts and fascia lata autografts (in Mihata’s studies the fascia lata autografts are approximately 8mm thick) may be superior to thin dermal allografts (3–4 mm), although a systematic review comparing dermal allograft and fascia lata concluded that both are acceptable [65–67]. A cohort study comparing SCR using fascia lata with or without mesh augmentation found improved healing, range of motion, and ASES scores in the mesh augmentation group [68]. Finally, LHBT has been introduced as a new and promising alternative for use in SCR in clinical and biomechanical studies [69, 70].

Although SCR appears to be a promising treatment based on early clinical results, it is a technically demanding operation that also requires intensive postoperative rehabilitation. Further high-quality studies are warranted to determine long-term results, as well as its effects on subsequent reverse total shoulder arthroplasty outcomes.

## Tendon Transfers

Over 30 years ago, Gerber introduced tendon transfers as a joint-preserving treatment option of irreparable rotator cuff tears for younger, active patients [71•]. Tendon transfers improve joint function and decrease pain by restoring force couples in the shoulder. Additionally, the transfer of muscle-tendon units potentially provides a vascularized autograft effect and powered tendon fibers, although the expected strength of the transferred tendon unit is at best one level lower compared to that of native function [17]. Several tendon transfers have been described including latissimus dorsi (LD), lower trapezius (LT), and pectoralis major (PM), with tendon selections often based on the location of the cuff tear. While these procedures were initially performed open, most can now be performed arthroscopically or with arthroscopic assistance [72, 73].

### Latissimus Dorsi

First described by Gerber in 1988, latissimus dorsi tendon transfer (LDTT) has historically been the most commonly used tendon transfer to treat irreparable posterosuperior cuff tears in young, active patients without glenohumeral osteoarthritis [71•]. In this procedure, the latissimus dorsi (LD) tendon is transferred from its insertion on the lesser tuberosity to the greater tuberosity, converting the LD from an internal rotator to an external rotator. This essentially replaces the posterior force couple of the infraspinatus and teres minor, which is counterbalanced by the deltoid and an intact subscapularis [74]. Although LDTT was originally performed as an open procedure, several studies have described arthroscopic and arthroscopic-assisted LDTT [75, 76].

Existing literature suggests LDTT is an effective treatment option to decrease pain in patients with irreparable posterosuperior cuff tears in the short and mid-term [77, 78]. Gerber et al. reported 74% good or excellent clinical results at mean 10-year follow-up [79]. In a more recent follow-up study, the same authors found that positive clinical results were maintained beyond 10-year follow-up after open LDTT; however, degenerative changes were present on follow-up radiographs [78]. El-Azab et al. also reported good pain relief and improvement in function and strength in 93 patients who underwent LDTT at a mean follow-up of 9 years. The reported failure rate was 10%, with 4% of patients ultimately converting to RTSA [80•].

Short- and mid-term improvements in pain relief and shoulder function have also been reported in series following arthroscopic or arthroscopic-assisted LDTT in patients with irreparable cuff tears [75, 76]. Castricini et al. described a series of 27 patients with a mean age of 60 years who showed significant improvement in mean Constant and pain scores (*p*<0.05) at mean follow-up of 27 months after undergoing arthroscopic-assisted LDTT. Additionally, they reported no significant progression in glenohumeral osteoarthritis or proximal humeral head migration at this point [75^•^]. Grimberg et al. described significant improvements in preoperative to postoperative mean Constant scores, range of motion, and subjective shoulder values at mean follow-up of 29 months (*p*<0.001) in a series of 55 patients with irreparable cuff tears who underwent arthroscopic-assisted LDTT. No significant progression in osteoarthritis or in acromiohumeral distance (AHD) was noted radiographically, although four tendon ruptures were reported at 1-year follow-up [81].

Despite these positive outcomes, functional improvement likely depends on patient selection. Numerous studies have reported worse outcomes in patients with concomitant subscapularis tears, fatty infiltration of teres minor, pseudoparalysis, and passive flexion or abduction less than 80 degrees [82–84]. Subscapularis tears in particular have consistently been associated with worse outcomes. Werner et al.’s cadaveric biomechanical study demonstrated that subscapularis dysfunction impacts centering of the humeral head during abduction and forward flexion, greatly increasing the risk of anterior subluxation [85]. This has led several authors to propose complete subscapularis tear as an absolute contraindication to LDTT for the treatment of irreparable posterosuperior cuff tears [83, 86].

Reported complications included progression of glenohumeral osteoarthritis, difficulty retraining the transferred tendon, and tendon rupture [87, 88]. Petriccioli et al. observed progression of glenohumeral osteoarthritis in 33% of patients who underwent arthroscopic-assisted LDTT for irreparable posterosuperior cuff tears at mean follow-up of 36 months [89]. Through electromyography study, Iannotti et al. showed that patients with poor clinical outcomes had difficulty retraining LD contraction, as none of the patients with poor outcomes were able to achieve synchronous, in-phase LD contraction during active external rotation [87].

Studies directly comparing outcomes following LDTT (open or arthroscopic-assisted) to other surgical options discussed in this review remain limited. As previously mentioned, a retrospective cohort study showed no difference in postoperative functional scores, active forward elevation, or external rotation at 2 years in those undergoing partial repair versus LDTT [26]. In a recent prospective randomized trial comparing patients treated with arthroscopic-assisted LDTT versus SCR, both cohorts experienced short-term clinical improvement. At 31 months follow-up, while both groups reported significant improvement in Constant, ASES, WORC, and VAS scores (*p*<0.01), the SCR cohort saw greater improvements in ASES (*p*=0.07) and Constant (*p*=0.008) scores. Additionally, in patients with pseudoparalytic shoulders, the SCR group had greater clinical success rates (92% vs. 45%, *p*=0.011). On the other hand, the LDTT group had significantly improved radiographic results as defined by AHD (*p*=0.006) [90•].

### Lower Trapezius

Originally described by Elhassan et al. in 2009, lower trapezius tendon transfer (LTTT) has emerged as an alternative to LDTT in treating irreparable posterosuperior cuff tears [91]. Initially, this procedure was indicated for patients with paralytic shoulders who lacked external rotation. According to cadaveric and biomechanical studies, the LT is a more anatomic selection for tendon transfer compared to the LD, as its line of pull is nearly identical to that of the infraspinatus [92]. However, the LTTT is an indirect transfer as the lower trapezius tendon lacks enough amplitude to reach the greater tuberosity without an interposition graft [17]. Still, LTTT theoretically provides a more effective external rotation moment arm compared to that of LDTT as well as similar excursion and tension forces as the native infraspinatus [93]. This theoretically may lead to a more “in-phase” transfer that is easier for patients to retrain postoperatively [94].

Several studies describe short-term improvements in pain and shoulder function after LTTT for treatment of irreparable posterosuperior cuff tears. In Elhassan’s original series on patients with brachial plexopathies, all 111 patients with paralytic shoulders achieved external rotation improvement with a mean increase of 70 degrees [93]. Elhassan also reported on 33 patients who underwent LTTT with Achilles tendon allograft, finding that 97% of patients had significant improvement in pain, function, and range of motion at 4-year follow-up [95•]. Mean improvement in forward flexion, abduction, and external rotation were 50 degrees, 50 degrees, and 30 degrees, respectively. Despite these studies reporting promising results after LTTT, durability of this outcome is unclear as there are currently no studies with long-term outcomes.

Considerations that are reportedly associated with improved outcomes after LTTT include patients with minimal to no glenohumeral osteoarthritis, preoperative shoulder flexion greater than 60 degrees, and less than 2 years time elapsed from symptoms to presentation [94].

### Pectoralis Major

First described by Wirth and Rockwood in 1997, pectoralis major transfer is used to treat irreparable anterosuperior cuff tears involving the subscapularis by restoring the anterior force couple of the shoulder [96]. Several surgical techniques have been described, which vary with regard to both the amount of tendon used and the position of the graft relative to the coracoid process [74]. Although there are no studies directly comparing outcomes with varied graft placement, biomechanical studies have demonstrated that a subcoracoid pectoralis major transfer more accurately replicates the force vector of the native subscapularis [97, 98].

Several studies described consistent improvements in pain yet varied functional restoration following pectoralis major transfer. Elhassan et al. reported improvements in preoperative to postoperative Constant scores (41 to 61) after pectoralis major transfer; however, 7/22 patients experienced tendon transfer failure at 49 to 57 months of follow-up [99]. Resch et al. also reported increases in Constant scores from 22 to 54 at mean follow-up of 28 months [97]. Additionally, Jost et al. saw an increase in mean relative Constant score from 47% preoperatively to 70% postoperatively [100]. Despite describing improvements in pain and Constant scores, Gavriilidis et al. reported no significant improvement in range of motion in a series of 15 patients at mean follow-up of 37 months after pectoralis major transfer. Furthermore, pectoralis major graft rupture was visualized after MRI follow-up in 15% of available patients [101].

Overall, pectoralis major transfer appears to be most successful in alleviating pain in patients with isolated irreparable subscapularis tears without any anterior instability. Patients with anterior subluxation of the humeral head have been observed to have worse outcomes and higher rates of failure [99]. Although the theoretical risk of neurologic injury is high given the close proximity of the musculocutaneous nerve, the reported incidence of postoperative nerve palsy is low, as a recent systematic review reported only one axillary nerve and one musculocutaneous nerve palsy out of 195 tendon transfers [102]. Additionally, worse outcomes have been reported in patients with concomitant irreparable supraspinatus tears [103]. This is likely due to anterosuperior migration of the humeral head secondary to imbalanced force coupling from supraspinatus dysfunction [104].

## Reverse Total Shoulder Arthroplasty (RTSA)

RTSA is an established treatment option for irreparable cuff tears in older patients with lower baseline activity levels. Described by Grammont in 1985, RTSA was originally used to exclusively treat rotator cuff tear arthropathy [105]. With improvement in surgical technique and RTSA implant design, indications have expanded to include irreparable cuff tears with and possibly without glenohumeral osteoarthritis [105–107].

In 2004, Frankle et al. reported improvements in functional scores, pain scores, and range of motion at 33 months follow-up in 60 patients who underwent RTSA for massive rotator cuff tears with glenohumeral osteoarthritis; however, there was a complication rate of 17%, with 12% of patients requiring revision due to early mechanical implant failure [108]. Analysis of these failures led to a change in implant design and surgical technique. A subsequent prospective study of 96 patients undergoing RTSA by the same group showed clinical improvement in pain and function while also demonstrating a reduced complication rate (9.4%) with no cases of early mechanical failure. Since then, numerous studies from different groups have also reported positive clinical outcomes following RTSA in patients over the age of 65 with irreparable rotator cuff tears and glenohumeral osteoarthritis at mid-term follow-up [106, 109–111].

Recent studies have also reported positive short- and mid-term clinical results in patients undergoing RTSA for irreparable cuff tears without glenohumeral osteoarthritis [107, 112•]. Mulieri et al. reported positive clinical outcomes in a series of 60 patients who underwent RTSA even in the absence of articular cartilage degeneration, with significant improvements in ASES, SST, and SF-36 scores as well as VAS pain scores and range of motion; however, they also reported a complication rate of 20% [107]. Recently, Frankle et al. reported significant improvement in range of motion, ASES, SST, and VAS scores at minimum 2-year follow-up in 92 patients over 65 years old with irreparable cuff tears and no glenohumeral osteoarthritis [113]. Hartzler et al. also described positive outcomes in functional and pain scores and range of motion in 74 patients without glenohumeral osteoarthritis who underwent RTSA for irreparable cuff tears; however, a complication rate of 17% was reported at minimum 2-year follow-up. Authors identified independent risk factors associated with poorer outcome after RTSA for this indication including higher preoperative functional status (SST ≥ 7), young age (<60 years old), and preoperative upper extremity neurologic dysfunction [112•]. Similarly, Werner et al. demonstrated that higher preoperative ASES scores were associated with worse functional improvement after RTSA in patients without glenohumeral osteoarthritis [114].

The role of RTSA in treating younger patients with irreparable cuff tears remains controversial given high patient activity levels, concern for implant longevity, and high complication rate of revision RTSA [115, 116]. Several studies have described positive short-term clinical outcomes [117, 118]. Muh et al. reported that 81% of patients under the age of 60 who underwent RTSA for irreparable cuff tears were very satisfied or satisfied at 36.5 months with significant improvements in pain and functional scores. Authors reported a complication rate of 15% [117]. Additionally, Samuelsen et al. reported a high patient satisfaction rate (90%) and improved functional and pain scores in patients under 60 years old at 3 years follow-up [118]. Yet, in both of these studies, indications for RTSA also included cuff tear arthropathy, failed primary total shoulder arthroplasty, and rheumatoid arthritis. Ernstbrunner et al. recently reported on long-term outcomes at mean follow-up of 11.77 years after RTSA in 23 patients (under 65 years old) with irreparable cuff tears. They described subjective and objective clinical improvement at mean follow-up of 12 years; however, they also reported 39% complication rate, 17% revision rate, and 9% failure rate [119••].

Despite the potential clinical improvements following RTSA in younger patients with irreparable cuff tears, surgeons should remain cautious when considering this procedure in a young, active patient given the high complication rate and known technical challenges associated with revision RTSA [120].

## Conclusions

Although the last several years have seen technical advances and innovations in the treatment of massive irreparable rotator cuff tears, there is still no clear consensus on a superior operative intervention. Thus, tailoring patient needs and characteristics to the chosen intervention is important to optimize the chance of success. Based on the current evidence, partial rotator cuff repair may be a good option for patients with lower preoperative function and may even produce results similar to more invasive interventions. Subacromial balloon spacers are a novel technique that appears to work best for patients without existing arthritis and with preserved passive range of motion, though long-term studies will be essential to better define indications. Superior capsule reconstruction has demonstrated excellent results in the hands of surgeons experienced with this complicated technique, and may even be an option for patients with pseudoparalysis, but it appears to be a poorer option for those with irreparable subscapularis tears. Tendon transfers of the LD and LT for irreparable posterosuperior tears and PM for anteroinferior tears may be reasonable options for young patients with high preoperative functional statuses. For older patients with low-demand lifestyles, RTSA is an effective treatment option. The role of RTSA in younger patients and in those without glenohumeral osteoarthritis remains controversial. Age less than 60, higher preoperative functional scores, and preoperative upper extremity neurological dysfunction have been associated with worse outcomes in these patient populations. Finally, much of the evidence discussed in this review comes from level III or IV studies, with few high-quality randomized controlled trials comparing various treatment methods. As such, further studies are required to further define treatments for these challenging injuries.
